# Effect of Two Educational Models including Face-to-Face and Training Pamphlets for Disease Control in Asthmatic Patients

**Published:** 2020-01

**Authors:** Vahideh Shariati, Reza Basiri, Farzaneh Iravani, Habibollah Esmaily, Reza Farid Hosseiny, Farahzad Jabbari Azad

**Affiliations:** 1 Specialist of Internal Medicine, Internal Department, Ghaem Hospital, Mashhad University of Medical Sciences, Mashhad, Iran,; 2 Lung Diseases Research Center, Mashhad University of Medical Sciences, Mashhad, Iran,; 3 Allergy Research Center, Mashhad University of Medical Sciences, Mashhad, Iran,; 4 Rheumatic Diseases Research Center, Mashhad University of Medical Sciences, Mashhad, Iran.

**Keywords:** Asthma, Disease control, Pamphlet, Face-to-face teaching, Asthma control test

## Abstract

**Background:**

Asthma is a common condition in which the patient requires self-management and teaching programs that lead to reduced prevalence and mortality. The main aim of this study was to improve the management knowledge of the disease through the use of educational tools, pamphlets and face-to-face lecture, concurrent with evaluating and comparing its effectiveness in response to treatment.

**Materials and Methods:**

In this study, 82 asthmatic patients were enrolled. Training necessary to control the disease and use of drugs were provided to patients in one group by pamphlets (39 patients) and the other by face-to-face education (43 patients). After a month, Disease control examination and Asthma Control Test (ACT) scores were evaluated and compared.

**Results:**

The mean age of participants was 39.12±14.25 years. There was no significant difference between the two groups in age, gender and education (P> 0.05) and no significant difference in asthma control between the two groups before the intervention (P = 0.065). The overall asthma control score in the pamphlet was increased from 15.43±4.99 at baseline to 20.58±4.47 in the assessment after one month education (P <0.001) and in face-to-face training an overall score was increased from 13.27±5.39 to 21.95±2.77 (P <0.001). After one month education, asthma control score was increased 5.23 ± 6.88 in pamphlets group and 8.9 ± 6.32 in face-to-face group (P = 0.014).

**Conclusion:**

Evaluation of both educational methods showed face-to-face training is more efficient.

## INTRODUCTION

Asthma is one of the most common chronic diseases all over the world (1–3), and about 300 million people are affected by asthma. It is expected that by 2025, 100 million asthmatic patients will be added to this amount (4). The global prevalence rates of physician diagnosed asthma and self-reported asthma in adults were 4.3 and 4.5%, respectively and varied by about 21-fold. Amongst data from 70 countries and Australia has the highest rate of physician diagnosed asthma (5). The researcher expressed that the prevalence of asthma has increased in rich countries, but at the moment it seems to be steady (1). They also reported that its prevalence in low-income countries is low; however it is doubted, as with respect to living conditions, exposure to air pollution and poor health statues it seems under diagnosed (6).

Asthma disease affects the living and social activities of patient’s life and limits their physical activity that might lead to psychological problems. The results of some studies relating to quality of living in patients with asthma indicate poor quality of life in these patients (7–9). Management of asthma requires daily self-management associated with acquisition of knowledge and related skills. Self-management is any behavior that people with asthma and their family members should do to reduce the effects of this chronic disease including diet therapy, along with a complex cognitive behavior, self-monitoring and linking between diet therapy and clinical symptoms (10).

Training programs can reduce incidence of the disease, mortality of patients, cost of treatment and improve the quality of life of these patients. In the field of self-management of asthma, considerable research has been done and many of them showed that the main cause of failure in the treatment of asthma is drug disobedience by patients and taking the wrong medication (11, 12).

Training can be done face-to-face by the therapist, or indirectly by a variety of educational pamphlets and videos but operating the most efficient and comprehensive method in patients is the key point (13).

Pamphlets are informative published subjects containing training information. Designing and developing educational pamphlet is very simple and low cost and can be widely distributed among them (14).

On the basis of mentioned pamphlets properties and the requirement for such an efficient, long-term educational instrument to teach the asthmatic patients, in this study the effectiveness of correct training of consumption of medicine and controlling environmental stimulation of asthma evaluated by two methods namely face-to-face method by the therapist and education by means of training pamphlets, was evaluated.

## MATERIALS AND METHODS

### Enrollment and sample size

The study began on June 15, 2016 and 82 patients that were older than 12 years, and after obtaining consent, referred to the Clinic of Allergy and Lungs and enrolled. This study was done in Allergy Research Center and Clinical Immunology and Allergy Clinic. Demographic data like age, gender, education level, severity of the disease and the state of patients was assessed before and after training. Asthma was confirmed by history, physical examination and spirometry and for allergy confirmation skin prick test was performed as well; at the end participations were randomly divided into two groups.

### Inclusion and exclusion criteria

We recruited asthmatic patients which were approved by a physician via spirometry or skin test. And as this clinical trial does not have any intervention, it does not have exclusion criteria and the patients were selected alternately.

### Study Design

At first, pamphlets were prepared about asthma and its irritants, how to use various sprays and something about food allergies.

In one group the mentioned pamphlets were given to patients and in the other group therapist trained the patients or their families face-to-face and offered the necessary explanations in 15 minutes.

A month later, patients referred again and therapist evaluated the control of the disease based on clinical examination and Asthma Control Test (ACT) form. ACT is a short and simple tool and consists of 5 general questions on asthma disease and duration of work, school or home disruption because of asthma, frequency of dyspnea, sleep disorders, consumption of life-saving medicines and the rate of asthma control according to the patient’s opinion. All questions were evaluated based on the past 4 weeks criteria and each question was assigned a score between 1 and 5 and at the end, the higher the score the better control of the disease.

### Statistical analysis

Statistical analysis was performed using SPSS 16 and P value of less than 0.05 was considered statistically significant.

### Sample size

The sample size was determined according to the same article by Kotwani et al. with specificity of 95% and sensitivity of 80% (15). The minimum sample size was 40 in each group and since there was the possibility of dropping samples, taking into account 20% drop, the sample size was considered as 50 in each group.

$$\text{N}=\frac{{\left(1/96+0/84\right)}^{2}\left(0/1^{2}+0/15^{2}\right)}{{\left(0/89-0/81\right)}^{2}}\approx 40$$

## Ethical considerations

The study protocol was approved by the Ethics Committee of Mashhad University of Medical Sciences and an informed consent was obtained from each participant or their legal representative (Code: IR.MUMS.REC.1391.401)

## RESULTS

In this study 82 asthmatic patients were evaluated and divided in two groups including pamphlet group (39 patients) and face-to-face training (43 patients) group.

The average age of patients participating in this study was 39.12±14.25 (12–79) years. In the study of each group individually, the average age was 36.87±14.28 years in pamphlet group and in face-to-face training group it was 41.16±14.08 years (P = 0.175) (Table. 1)

**Table 1.** Characterization of underlying homogeneity of subjects in both face-to-face learning and pamphlets groups

**Parameters**		**Pamphlet Group** **N=39**	**Face-to-Face Group** **N=43**	**P Value**
**Age, (Mean ± SD)**		36.87±14.28	41.16±14.08	P=0.175*
				t=−1.36
				df=79
**Sex, No. (Percentage)**	Male	12 (30.8)	20(46.5)	X2=2.13
	Female	27 (69.2)	23(53.5)	P=0.144**c
**Education, No. (Percentage)**	The illiterate and primitive	18(46.15)	19(44.18)	X2=0.032
	Above the primitive	21(53.85)	24(55.82)	P=0.858**
	Under 18 years	2(5.1)	3(6.97)	X2=0.322
**Range of Age, No. (Percentage)**	18 to 50 years	30(76.9)	29(67.44)	0.57***
	Higher than 50 years	7(18)	11(25.59)	

Gender distribution of patients was not significant between the two groups; in the pamphlet group, 30.8% were male and in the face-to-face group 46.5% were male (P = 0.144). There was no significant difference between the two groups in terms of distribution of education level and age range (P = 0.858 and P = 0.634).

There was no significant difference between the two groups before training in terms of asthma control (P = 0.065) and showed that the two groups were homogeneous in terms of asthma severity.

**Figure 1.**
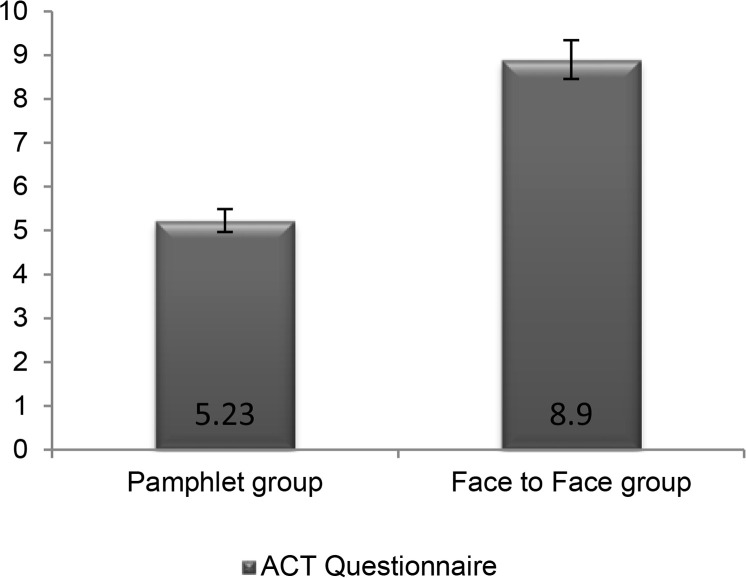
Comparison of the quality of asthma control between two groups before and after pamphlet training and face-to-face training (P <0.001)

The total score in the pamphlet group increased from 15.43 ± 4.99 at the beginning of the study to 20.58 ± 4.47 in the evaluation one month later, and in the face-to-face training group the overall score increased from 13.27 ± 5.39 to 21.95 ± 2.77. Differences after the intervention compared to the situation prior to the intervention in both groups were statistically significant (P <0.001) (Figure 1).

Compared before and after one-month training, it was observed that in pamphlets group the score increased about 5.23±6.88 points and in face-to-face training increased at a rate of 8.9±6.32, representing a better improvement in the state of patients in the face-to-face group (P = 0.014). Considering the basis of age in the age categories under 18 and over 50 years, there was no significant difference between the two groups, respectively (P = 0.14 and P = 0.86), but in the age group 18–50 years the state of patients in face-to-face training was significantly better (P = 0.005) (Table 2). Comparison within the three age categories in any of the two pamphlets (P = 0.153) and face-to-face training groups (P = 0.388) was not statistically different.

**Table 2.** Comparison of changes in control of asthma between the two pamphlets and face-to-face groups before and after training divided by age, gender and level of education

**Categories**		**Pamphlet Group**		**Face-to-Face Group**		P Value*

		**Number**	**mean±SD**	**Number**	**mean±SD**	
**Total**		39	5.23±6.88	43	8.9±6.32	0. 14
**Age**	Under 18 years	2	14±4.24	3	10.33±1.15	0.223
	18 to 50 years	30	4.43±7.22	29	9.62±6.46	0.005
	Higher than 50 years	7	6.14±3.89	11	6.63±6.56	0.86
**Sex**	Male	12	5.83±7.32	20	8.55±5.78	0.253
	Female	27	4.96±6.81	23	9.21±6.87	0.033
**Level of Education**	The illiterate and Primitive	18	6.55±5.51	19	8.26±6.5	0.396
	Above the primitive	21	4.09±7.83	24	9.41±6.26	0.015

Data represent that on the basis of sex evaluation, improvement in the state of patients, in men there was no significant difference between the two groups (P = 0.86), but in women, in face-to-face training group, state of patient improvement was significantly better (P = 0.033).

Assessment within the groups showed that between the two sexes neither pamphlets group (P = 0.721) nor face-to-face training group (P = 0.734) was not statistically different.

Evaluation according to the level of education, patients were divided into three groups including the group with the level of primary education and illiteracy and higher than primary. There was no significant difference between the two training of pamphlets and face-to-face method in the group with the level of primary and illiterate education (P=0.396), but asthma control in the higher elementary group compared to face-to-face group and pamphlet group showed significant improvement (P=0.015).

Intra-group comparison using t-test showed that there was no significant difference between the two levels of education in the two pamphlet and face-to-face groups, respectively (P=0.272 and P=0.559).

We used covariance analysis to evaluate the interferer variables such as gender, sex and ACT before the intervention (Table 3). The results of covariance analysis showed that by controlling interfering parameters such as gender, age and score, before the intervention, outcome of training in face-to-face group showed approximately 2.069 higher score than pamphlet group.

**Table 3.** Analysis of covariance result in both face-to-face and pamphlets group on the changes of ACT score

**Parameters**	**Type**	**The regression factor**	**Standard Error**	**T**	**P-value**
**Sex**	Male	−0.511	0.781	0.654	0.515
	Female	0	-	-	-
**Group**	Face-to-Face Group	2.069	0.789	2.622	0.011
	Pamphlet Group	0	-	-	-
**Pretest ACT**	0.296	0.073	4.037	<0.001	
	Under 18 years	2.58	1.684	1.53	0.129
	18 to 50	−0.398	0.985	−0.404	0.687
	Higher than 50 years	0	-	-	-

## DISCUSSION

In this study both methods made the significant difference after the intervention compared to the situation before (P<0.001) and both types of training improved control of asthma patients. However comparison of changes in control of asthma showed that the state of patients in the face-to-face group was significantly better (P=0.14, t=2.48, df=77).

Mancuso et al. also studied 296 patients with asthma in terms of evaluating the effectiveness of two different training methods. By examining the quality of patients life during the 4th, 12th and 16th weeks after training, they showed that education by providing pamphlets called Asthma science, Peak ticking training, asthma brochures, the Asthma management booklet and inhalation and telephone training are less effective than contact with physicians. Of course, this study found that both methods had high efficacy and made a significant change in the status of patients, but the results of this study confirmed that the pamphlet alone could not replace face-to-face communication and direct physician contact with patient (16).

About the positive impact of pamphlets, Gawwad et al. also examined the usage of pamphlets as a training tool for teaching asthma control to school staff in 2007 in Riyaz city. In this study, 297 school staff were selected at various levels of pre-test. The results showed that only 5.7% of the staff had previously been trained in asthma. In the post test, this percentage increased significantly to 83.9 and 68.6%, respectively. The results showed that a simple educational intervention using the pamphlet and displaying the use of spray and peak flow meters was significantly effective in raising the information and management of asthma in school staffs (13).

Poureslami and coworkers, in a study in 2012, compared the two educational and general video training by similar content but with two scientific and idiomatic approaches with a pictorial pamphlet. A total of 92 asthmatic patients were divided into 4 groups: seeing one film, both films and study of the pamphlet. Patients’ knowledge of asthma stimuli, symptoms, spray technique, and reporting rates were assessed by physicians. In this study, level of knowledge on asthma symptoms, method of using spray and understanding the orders of the physician from the beginning of the study to 3 months later was significantly increased in all participants. This improvement was more pronounced especially in the group seeing both educational and general films (17).

On the other hand, in a study by Braido et al. in 2011, comparing face-to-face training and written teaching, it was shown that the relationship between doctor and patient was overlooked in the long run. Finally in the group who received writing training, asthma control was better and severity of asthma was reduced. These findings also show the sustainability of teaching by pamphlet compared to face-to-face training in the long run (18).

Our results show that there was no significant difference in the age categories under 18 and over 50 years (respectively P = 0.14 and P = 0.86), but in the age categories 18 to 50 years face-to-face training was better than pamphlets (P = 0.005). In men, there was no significant difference between the two methods of education (P = 0.86), but face-to-face training was more effective in women (P = 0.033). There was no significant difference in the effectiveness of two types of education in patients with elementary and illiterate level (P = 0.396), but faculty members were more effective in face-to-face training (P = 0.015).

We expected that the pamphlets education would be more effective in higher literacy level group than face-to-face training, but contrary to expectation, this method in illiterate and primitive group had similar performance in face-to-face higher literacy training group.

It was also expected that the effectiveness of the pamphlet at a young age (18 to 50 years) and female gender would probably be higher for study, but contrary to expectation, face-to-face education was more effective. However, these results were obtained in single-variable analysis, while using covariance analysis differences between age groups were not significant. One of the reasons for lower efficiency of the pamphlet method in this study may be the incomprehensibility of the pamphlet for the general public training.

As Sarma et al. in a study in Australia reported the availability and understandability of asthma related pamphlets, considering Grade 1 as the most comprehensible to Grade 12 for the hardest; one third of the patients got Grade over 9 and two thirds Grade 8 or higher. These results showed that a small percentage of the pamphlets available for the educational project were legible and understandable (19).

The first limitation of this study was the small sample size and the second limitation was about the asthma control that was evaluated by a measurement tool named ACT questionnaire, which is part of its judgment and scoring based on the patient’s own opinion, which can impact the results of the study.

## CONCLUSION

In this study the efficiency of both educational pamphlet and face-to-face training in controlling symptoms of asthma patients has been shown. It can be inferred that using such an accessible and simple educational intervention, if properly designed with all necessary and needed information at the patient’s level, might be better for the asthma-related knowledge, management and even the attitude towards the disease. However, on the basis of our results we investigated that the effectiveness of face-to-face training was more than pamphlets, therefore this study is recommended to make use of face-to-face training by therapist and patients; with a greater emphasis on regular visits of patients by doctors. Although pamphlet is a permanent reminder; it can be read anytime and anywhere and also an auxiliary method for transmitting information to patients, especially for information that requires frequent repetition and practice.
